# Colon-Specific Delivery of Febuxostat via an Amino Acid Conjugate Enhances Anti-Colitis Efficacy and Limits Systemic Exposure

**DOI:** 10.3390/pharmaceutics18091187

**Published:** 2026-09-20

**Authors:** Suji Kim, Sanghyun Ju, Yunjin Jung

**Affiliations:** 1 College of Pharmacy, Pusan National University, Busan 46241, Republic of Korea; soozykim34@gmail.com (S.K.); jsh141002@naver.com (S.J.); 2 Research Institute for Drug Development, Pusan National University, Busan 46241, Republic of Korea

**Keywords:** febuxostat, colon-specific drug delivery, colitis, prodrug, drug repositioning

## Abstract

**Background/objectives:** Febuxostat (FBXT), a xanthine oxidase (XO) inhibitor, is a urate-lowering drug commonly used to treat gout. XO is also a promising therapeutic target for inflammatory bowel disease (IBD). This study aimed to improve the efficacy and safety of FBXT repurposed for the treatment of IBD. **Methods:** FBXT was conjugated with the acidic amino acids aspartic acid (Asp) and glutamic acid (Glu) via amide linkages to yield the colon-specific conjugates FBXT-Asp and FBXT-Glu. **Results:** The results revealed that amino acid conjugation decreased the distribution coefficient of FBXT from 1.451 to −1.217 for FBXT-Asp and −0.859 for FBXT-Glu. In parallel, conjugation retarded FBXT transport across the small intestinal wall, with a more pronounced effect observed with Glu conjugation than with Asp conjugation. The conjugates were converted to FBXT in cecal contents under a nitrogen atmosphere while remaining intact in pH 1.2 buffer and small intestinal contents. The cecal conversion percentages at 8 and 24 h were 7.1% and 22%, respectively, for FBXT-Asp and 17% and 54.8%, respectively, for FBXT-Glu. Consistent with these in vitro results, oral gavage of FBXT-Glu resulted in greater FBXT accumulation in the rat cecum than that observed with free FBXT. In rats with 2,4-dinitrobenzenesulfonic acid-induced colitis, oral gavage of FBXT-Glu at a dose equivalent to 20 mg of FBXT ameliorated colonic damage and inflammation. This effect was more potent than that of FBXT at the same dose or sulfasalazine (SSZ), a currently used anti-IBD drug. Additionally, FBXT-Glu did not produce detectable blood levels of FBXT, whereas FBXT was detected at concentrations of up to 6.7 μM at 2 h after oral gavage of FBXT at the corresponding dose. **Conclusions**: FBXT-Glu may serve as an anti-colitis agent with therapeutic and toxicological advantages over FBXT and could be a viable alternative to SSZ.

## 1. Introduction

Crohn’s disease (CD) and ulcerative colitis (UC) are the two primary, chronic, and relapsing types of inflammatory bowel disease (IBD). Although they share similar symptoms, they differ in the location and depth of bowel inflammation. Unlike CD, which causes transmural lesions (deep inflammation) and is associated with a higher risk of fistula formation in any part of the gastrointestinal (GI) tract, UC is characterized by continuous mucosal inflammation that is strictly limited to the rectum and colon [1]. The pathogenesis of IBD remains unclear. Current evidence suggests that IBD results from a chronic, multifactorial process driven by an inappropriate immune response to the gut microbiota in genetically susceptible individuals with immune dysfunction, impaired intestinal epithelial barrier function, and environmental triggers. These susceptible individuals fail to maintain tolerance to the normal gut microbiota, resulting in uncontrolled chronic inflammation of the intestinal wall [1,2].

Current anti-IBD pharmacotherapies aim to induce and maintain remission of inflammation and encompass a range of treatments, from traditional anti-inflammatory agents and immunosuppressants to targeted biologics and small molecules [3]. Key treatments include aminosalicylates, corticosteroids, immunomodulators, tumor necrosis factor-alpha (TNF-α) inhibitors, integrin receptor antagonists, interleukin (IL)-12/IL-23 inhibitors, Janus kinase inhibitors, and sphingosine-1-phosphate receptor modulators [3,4]. Targeted therapies provide satisfactory clinical efficacy in patients with IBD who are resistant or intolerant to conventional treatments. However, traditional therapies often have limited efficacy and can cause serious adverse effects during the long-term treatment required for IBD management. Moreover, targeted therapies still have medical and practical limitations [5]. Their adverse effects can be severe with prolonged administration, and biologics may induce antidrug antibodies that neutralize their therapeutic activity in patients receiving long-term treatment [5,6]. Biologic therapies are also associated with practical challenges, including financial burdens and poor patient adherence due to high medical costs and the need for parenteral administration [7,8]. Therefore, the development of small-molecule drugs with improved efficacy and reduced toxicity remains an unmet need in IBD treatment.

Xanthine oxidase (XO), an enzymatic form of xanthine oxidoreductase (XOR), contributes to XOR-mediated oxidation. It plays a key role in purine catabolism by sequentially oxidizing hypoxanthine to xanthine and xanthine to uric acid (UA) [9]. This enzymatic activity makes XO a target of the anti-gout drugs febuxostat (FBXT) and allopurinol, which inhibit XO and reduce blood UA levels [10,11]. During inflammatory processes, XO facilitates the production of superoxide radicals and nitric oxide in multiple organ systems, with the intestinal tract being a major site of this activity. The gut is among the human organs with the highest XO activity, which increases further under inflammatory conditions [12,13,14,15]. Beyond its role in generating harmful reactive species, emerging evidence identifies XO as a proinflammatory mediator that activates the NLRP3 inflammasome, a protein complex critical for the processing of proinflammatory cytokines such as IL-1β and IL-18 into their active forms [12]. Furthermore, XO-derived reactive oxygen species (ROS) and UA have been implicated in distinct mechanisms that promote NLRP3 activation and drive IL-1β overexpression. Additionally, the XO inhibitors FBXT and allopurinol can modulate inflammatory responses by inhibiting NLRP3 and NF-κB activation and reducing the production of ROS and proinflammatory mediators, including TNF-α, IL-1β, IL-6, IL-8, transforming growth factor beta 1, monocyte chemoattractant protein-1, and cyclooxygenase-2 [10,16]. Therefore, XO is considered a promising therapeutic target for IBD; its overexpression in the inflamed mucosa of patients with IBD may sustain intestinal inflammation by activating NLRP3 and promoting proinflammatory cytokine production [12]. Indeed, XO inhibitors have demonstrated anti-colitis activity in animal models of colitis [17,18,19,20].

To date, three XO inhibitors have been used clinically: allopurinol (approved in 1966), FBXT (approved in 2009), and topiroxostat (approved in 2013) [21,22]. Structurally, allopurinol is classified as a purine derivative, whereas FBXT and topiroxostat are nonpurine inhibitors. These nonpurine agents exhibit greater therapeutic potency and urate-lowering capacity than allopurinol [21,23]. Allopurinol is generally considered a safer first-line treatment for gout, whereas FBXT carries an FDA boxed warning regarding an increased risk of cardiovascular and all-cause mortality, particularly in patients with pre-existing cardiovascular disease [22,24]. However, recent clinical studies have provided evidence that the safety of FBXT is comparable to that of allopurinol, even in patients with pre-existing cardiovascular disease [25,26,27]. Because drug repositioning requires an acceptable established safety profile [28], these clinical findings support FBXT as a candidate, alongside allopurinol, for repurposing in conditions in which XO represents a therapeutic target.

To restrict the release of orally administered drugs to the lower GI tract, colon-specific drug delivery systems have been developed [28]. For the treatment of localized intestinal diseases such as IBD, this site-directed approach offers substantial therapeutic and safety advantages [29,30]. Delivering the active pharmaceutical ingredient directly to the inflamed mucosa while minimizing systemic exposure can enhance therapeutic efficacy and reduce systemic adverse effects [29,30].

Although FBXT has considerable potential as an anti-IBD drug, no previous studies have investigated its repurposing for IBD using a colon-specific drug delivery strategy. In this study, FBXT was chemically modified to act locally in the large intestine, with the aim of enhancing its therapeutic efficacy and safety for clinical application in IBD. To achieve this, the established colon-specific carriers [30], aspartic acid (Asp) and glutamic acid (Glu), were linked to the carboxyl group of FBXT via amide linkages, and the colon-specific properties of the resulting conjugates were subsequently evaluated using in vitro and in vivo assays. The conjugate exhibiting greater colon-targeting performance was pharmacologically evaluated in a rat model of 2,4-dinitrobenzenesulfonic acid (DNBS)-induced colitis and compared with free FBXT and the locally acting anti-IBD drug sulfasalazine (SSZ) [31]. Finally, the potential for systemic adverse effects was assessed by comparing the systemic absorption of the amino acid–FBXT conjugate with that of free FBXT after oral administration.

## 2. Materials and Methods

### 2.1. Materials

FBXT was isolated from commercially available Feburic^®^ tablets (80 mg/tablet; SK Chemicals, Seongnam, Republic of Korea). Briefly, the yellow outer film coating was mechanically removed, and the tablet core was pulverized into a fine powder using a mortar and pestle. The resulting powder was suspended in 1 M NaOH and centrifuged to remove insoluble substances. The supernatant was acidified with 1 M HCl, followed by partitioning with ethyl acetate (EA) and evaporation under reduced pressure. SSZ and DNBS were purchased from Tokyo Chemical Industry Co., Ltd. (Tokyo, Japan). Amino acid derivatives, including *L*-aspartic acid dimethyl ester (AspDME) hydrochloride and *L*-glutamic acid dimethyl ester (GluDME) hydrochloride, were purchased from Ambeed, Inc. (Arlington Heights, IL, USA). Triethylamine (TEA) was purchased from DUKSAN Pure Chemicals (Ansan, Republic of Korea), and thionyl chloride (SOCl_2_) was obtained from Fluka Chemika (Buchs, Switzerland). Solvents used for compound synthesis and high-performance liquid chromatography (HPLC) analysis were supplied by Junsei Chemical Co., Ltd. (Tokyo, Japan) and DAEJUNG Chemicals & Metals Co., Ltd. (Siheung-si, Gyeonggi-do, Republic of Korea). For mobile-phase preparation, potassium phosphate monobasic was purchased from Sigma-Aldrich Chemical Co., Inc. (St. Louis, MO, USA), and phosphoric acid (Extra Pure grade) was obtained from DAEJUNG Chemicals & Metals. Fluorescein isothiocyanate (FITC)-dextran (average molecular weight, 4000) was purchased from Sigma-Aldrich Chemical Co., Inc. Unless otherwise stated, all reagents and chemicals used in this study were of analytical or HPLC grade and were used without further purification. The final products were structurally characterized using a Nicolet iS50 Fourier-transform infrared (FT-IR) spectrophotometer (Thermo Fisher Scientific, Waltham, MA, USA) and an Avance NEO 500 proton nuclear magnetic resonance (^1^H-NMR) spectrometer (Bruker Corporation, Billerica, MA, USA). Mass spectra were acquired through electrospray ionization mass spectrometry (ESI-MS) using a 6530 Q-TOF mass spectrometer (Agilent Technologies, Santa Clara, CA, USA).

### 2.2. Synthesis of FBXT Conjugated with an Acidic Amino Acid

A solution of FBXT (316.3 mg, 1 mmol) in 30 mL of dichloromethane (DCM) was stirred for 10 min at 20–25 °C and then cooled in an ice bath. The reaction solvent was dried over anhydrous sodium sulfate before use. Subsequently, SOCl_2_ (72.6 μL, 1 mmol) was added dropwise. To prevent moisture exposure and hydrolysis during the synthesis of moisture-sensitive FBXT-COCl, all reactions were carried out using oven-dried glassware in a firmly sealed system. Furthermore, reagents, including SOCl_2_, were introduced using air-tight syringes through rubber septa to minimize exposure to ambient air. After stirring for an additional 10 min at 0 °C, the mixture was treated with GluDME hydrochloride (254.0 mg, 1.2 mmol) and TEA (557.5 μL, 4 mmol). After incubation for 1 h at 0 °C, the coupling reaction was quenched by adding 30 mL of an aqueous 5% (*w*/*v*) NaHCO_3_ solution. The organic layer was separated and washed three times with 0.1 M HCl, followed by 0.1 M NaOH. The DCM was subsequently removed under reduced pressure to yield a crude residue. The residue was then subjected to ester hydrolysis with 15 mL of 1 M NaOH under continuous stirring for 1 h at 20–25 °C. The mixture was subsequently acidified with 1 M HCl, and the aqueous phase was thoroughly extracted with EA. The combined organic layers were dried over anhydrous Na_2_SO_4_, filtered, and concentrated to dryness under reduced pressure to yield *N*-[2-(3-cyano-4-isobutoxyphenyl)-4-methyl-1,3-thiazole-5-carbonyl]-*L*-glutamic acid (FBXT-Glu) as a white powder. FBXT-Glu: M.W. 445.5; yield, 28%; mp, 108 °C; IR (Nujol mull, νmax, cm^−1^): 1708 (C=O stretch, carboxylic acid), 1635 (C=O stretch, amide); ^1^H-NMR (deuterated dimethyl sulfoxide [DMSO-d_6_], δ, ppm): 8.50 (d, J = 6.0 Hz, 1H), 8.24 (d, J = 2.4 Hz, 1H), 8.18 (dd, J_1_ = 8.4 Hz, J_2_ = 2.4 Hz, 1H), 7.38 (d, J = 9.0 Hz, 1H), 4.35 (m, 1H), 4.00 (d, J = 6.0 Hz, 2H), 2.59 (s, 3H), 2.30–2.45 (m, 2H), 2.08 (m, 1H), 1.89–2.05 (m, 2H), 1.00 (d, J = 6.0 Hz, 6H); ESI-MS (*m*/*z*): [M − H]^−^ = 444.1239.

*N*-[2-(3-Cyano-4-isobutoxyphenyl)-4-methyl-1,3-thiazole-5-carbonyl]-*L*-aspartic acid (FBXT-Asp) was synthesized using the same method, with AspDME hydrochloride substituted for GluDME hydrochloride. The product was obtained as a white powder. FBXT-Asp: M.W. 431.5; yield, 33%; mp, 129 °C; IR (Nujol mull, νmax, cm^−1^): 1698 (C=O stretch, carboxylic acid), 1645 (C=O stretch, amide); ^1^H-NMR (DMSO-d_6_, δ, ppm): 8.51 (d, J = 6.0 Hz, 1H), 8.24 (d, J = 2.0 Hz, 1H), 8.18 (dd, J_1_ = 8.5 Hz, J_2_ = 2.0 Hz, 1H), 7.37 (d, J = 9.0 Hz, 1H), 4.70 (m, 1H), 4.00 (d, J = 6.0 Hz, 2H), 2.65–2.85 (m, 2H), 2.58 (s, 3H), 2.08 (m, 1H), 1.00 (d, J = 6.0 Hz, 6H); ESI-MS (*m*/*z*): [M − H]^−^ = 430.1087.

### 2.3. HPLC Analysis

Samples were analyzed using a Gilson HPLC system equipped with a Gemini NX-C18 column (150 × 4.6 mm, 5 μm; Phenomenex, Torrance, CA, USA) for chromatographic separation. HPLC analysis was performed at ambient temperature. Isocratic elution was performed using a 50:50 (*v*/*v*) mixture of acetonitrile and 10 mM potassium phosphate buffer, with the aqueous buffer adjusted to pH 3.0 using phosphoric acid before mixing. The mobile phase was delivered at a constant flow rate of 1 mL/min. Analytes were detected at 315 nm using a Gilson 151 variable UV detector with an absorbance unit full scale setting of 0.01. The retention times were 2.7, 2.8, and 5.6 min for FBXT-Asp, FBXT-Glu, and FBXT, respectively. The limits of detection for the conjugates and FBXT in biological samples ranged from 0.95 to 1 μM. The linear equations for the standard calibration curves are provided in Supplementary Information S1.

### 2.4. Distribution Coefficient, Chemical Stability, and Intestinal Permeability

The partitioning behavior of FBXT and its conjugates (FBXT-Asp and FBXT-Glu) was evaluated using an *n*-octanol/isotonic phosphate buffer (pH 6.8) system according to a previously reported method [32]. FBXT was dissolved in 10 mL of buffer-saturated *n*-octanol, whereas the conjugates (FBXT-Asp and FBXT-Glu) were dissolved in 10 mL of *n*-octanol-saturated isotonic phosphate buffer (pH 6.8). Each solution was then mixed with 10 mL of the opposite phase. After orbital shaking for 12 h at 200 rpm, the samples were allowed to equilibrate at 25 °C for 4 h to ensure complete phase separation. The concentrations of the test compounds in the aqueous phase were measured at 315 nm using a UV–Vis spectrophotometer (Shimadzu, Kyoto, Japan). The log D_6.8_ values were calculated using the equation below, where C_0_ represents the initial concentration of each test compound, and C_W_ and C_OC_ represent the equilibrium concentrations of the test compound in the aqueous and *n*-octanol phases, respectively.

$$logD_{6.8}=\log(C_{Oc}/C_{W})=log[{(C}_{0}-C_{w})/C_{w}]$$

Conjugate stability was assessed using HPLC by monitoring time-dependent changes in the concentrations of the conjugates during incubation in an acidic buffer (pH 1.2 HCl–NaCl) and in 10% small intestinal contents suspended in a pH 6.8 isotonic phosphate buffer (1.0 mM) at 37 °C for 10 h. The stability and log D_6.8_ values of the conjugates were measured in triplicate.

For intestinal permeability studies, male Sprague–Dawley (SpD) rats were fasted for 24 h with ad libitum access to water. Following euthanasia by CO_2_ asphyxiation, approximately 6 cm segments of the jejunum were promptly excised. These jejunal segments were used to investigate the transport of FBXT (0.5 mM) and its conjugates (FBXT-Asp and FBXT-Glu; 0.5 mM). The excised tissues were gently flushed with sterile, prewarmed (37 °C) Krebs buffer (120.0 mM NaCl, 5 mM KCl, 2 mM CaCl_2_, 1 mM MgCl_2_, 5.5 mM HEPES sodium salt, and 1.0 mM D-glucose), which had been filtered through a 0.45-μm membrane before use. The tissue was carefully everted using forceps and a glass rod, after which the exposed mucosal surface was rinsed with the same buffer. One end of each segment was ligated, and 600 μL of fresh Krebs buffer was introduced into the internal compartment. The resulting everted sac was then immersed in a 15 mL conical tube containing 8 mL of Krebs buffer. The liquid levels in the two compartments were carefully matched to prevent hydrostatic pressure differences. The system was incubated at 37 °C. At predetermined intervals, 50-μL aliquots were collected from the interior of the sac and immediately replaced with an equal volume of fresh buffer. For HPLC analysis, proteins were precipitated by adding 450 μL of methanol to each aliquot. The mixtures were vigorously vortexed and centrifuged at 10,000× *g* at 4 °C for 10 min. The resulting supernatants were passed through a 0.45-μm filter before quantification. To assess intestinal barrier integrity, an additional assay was performed using FITC-dextran (100 μM) co-applied with either FBXT or its conjugates (0.5 mM) to the apical side under the same experimental conditions, as described in Supplementary Information S2.

### 2.5. Animals

Seven-week-old male SpD rats were obtained from Samtako Bio Korea (Osan, Gyeonggi-do, Republic of Korea) and used for the in vivo studies. The animals were housed in the animal facility of Pusan National University (Busan, Republic of Korea) under controlled conditions, including a 12 h light/dark cycle, constant ambient temperature, and controlled humidity. All animal procedures were conducted in accordance with the guidelines of the Institutional Animal Care and Use Committee of Pusan National University and were reviewed and approved by the committee before the experiments were initiated (approval no. PNU-2025-0717; approved 3 December 2025).

### 2.6. Incubation of Drugs in the Small Intestinal and Cecal Contents of Rats

To assess the colon-specific activation of the conjugates, luminal contents from the small intestine and cecum of CO_2_-euthanized male SpD rats (250–260 g) were collected via laparotomy. Suspensions (20%, *w*/*v*) of the small intestinal luminal contents were prepared using isotonic phosphate buffer (pH 6.8). To maintain anaerobic conditions, both ends of the cecal segments were clamped before excision, and the segments were immediately transferred to an anaerobic chamber bag (AtmosBag; Sigma-Aldrich, St. Louis, MO, USA) filled with nitrogen. The cecal luminal contents were then collected and suspended in isotonic phosphate buffer (pH 6.8) to prepare 20% (*w*/*v*) suspensions. The FBXT conjugates (1 mM), dissolved in 5 mL of isotonic phosphate buffer (pH 6.8), were mixed with 5 mL of the cecal or small intestinal suspensions and incubated at 37 °C. For incubation with the cecal contents, the buffers were bubbled with nitrogen gas for 10 min before use.

At predetermined time points, 300-μL samples were collected and extracted with 0.3 mL of EA. The mixtures were mechanically agitated for 5 min and centrifuged at 10,000× *g* at 4 °C for 10 min. Subsequently, a 200-μL aliquot of the EA extract was evaporated to dryness using a centrifugal vacuum concentrator (CVE-2200; EYELA, Tokyo, Japan). The residues were redissolved in 200 μL of methanol and filtered through a 0.45-μm membrane before HPLC quantification. To quantify the liberated FBXT, the samples were diluted 10-fold by mixing 50 μL of each sample with 450 μL of methanol, and 20 μL of the resulting solution was injected into the HPLC system.

### 2.7. Analysis of Drug Concentrations in Blood and Cecum

Before the experiments, male SpD rats were fasted for 24 h with ad libitum access to water. For oral gavage, 1.0 mL suspensions of FBXT (20 mg/kg) and FBXT-Glu (28 mg/kg, equivalent to 20 mg/kg FBXT) were freshly prepared in phosphate-buffered saline (PBS; pH 7.4). At 2 and 6 h after administration, blood was collected from separate groups of rats at each time point via cardiac puncture and centrifuged at 10,000× *g* at 4 °C for 10 min to obtain approximately 0.3 mL of plasma. To quantify plasma concentrations of FBXT and FBXT-Glu, 100 μL of plasma was deproteinized with 900 μL of methanol. After vortexing for 5 min and centrifugation, the supernatant was filtered through a 0.45-μm membrane, and 20 μL of the filtrate was injected into the HPLC system.

To quantify cecal drug concentrations, cecal contents collected at 2 and 6 h after oral gavage were homogenized in isotonic phosphate buffer (pH 6.8) to prepare a 10% (*w*/*v*) suspension. A 100-μL aliquot of the suspension was mixed with 900 μL of methanol and mechanically agitated for 5 min. The samples were then centrifuged at 10,000× *g* at 4 °C for 10 min. The resulting supernatant was filtered through a membrane filter, and 20 μL of the filtrate was injected into the HPLC system for analysis.

### 2.8. DNBS-Induced Rat Colitis

A chemically induced colitis model was established in male SpD rats (body weight, 250–260 g) as previously described [31]. Before DNBS administration, the rats were fasted for 24 h with ad libitum access to water and then anesthetized. Anesthesia was induced in a dedicated chamber with 3% isoflurane (Hana Pharm, Hwaseong, Republic of Korea) delivered in oxygen at a flow rate of 1 L/min. For maintenance of anesthesia during subsequent procedures, the isoflurane concentration was reduced to 2.5% using a Small Animal O_2_ Single Flow Anesthesia System (LMS, Pyeongtaek, Republic of Korea). After adequate anesthetic depth was confirmed by the absence of the pedal withdrawal reflex, a rubber cannula with an outer diameter of 2 mm was inserted rectally and advanced approximately 8 cm into the colon toward the splenic flexure. DNBS (58 mg) dissolved in 0.4 mL of 50% aqueous ethanol was then carefully administered through the cannula.

### 2.9. Evaluation of Anti-Colitis Effects

To evaluate the therapeutic efficacy of the test compounds against DNBS-induced colitis, the in vivo experiments were conducted over a 10-day period, as shown in Supplementary Information S3A. Following the induction of colitis, the animals were allowed a 3-day stabilization period before treatment. The rats were randomly assigned to treatment groups. Two separate animal experiments were conducted to evaluate anti-colitis efficacy. In both experiments, the drugs were suspended in PBS to a final volume of 1 mL and administered by oral gavage once daily for six consecutive days. The first experiment comprised four groups (*n* = 5 per group), whereas the second independent experiment comprised five groups (*n* = 5 per group). The treatment groups and corresponding doses are presented in Supplementary Information S3B,C.

At 24 h after the final administration, all animals were humanely euthanized for sample collection and therapeutic assessment. Macroscopic colonic injury was quantified using a modified numerical scoring system based on structural lesions, strictures, and the extent of inflammation (Supplementary Information S4). To minimize bias, colonic lesions were evaluated independently by four researchers who were blinded to group allocation Additionally, the terminal 4 cm segment of the colon was used to determine myeloperoxidase (MPO) activity as previously described [33]. Distal colon samples were sectioned and homogenized in 0.5% hexadecyltrimethylammonium bromide buffer (pH 6.0) at a tissue concentration of 100 mg/mL using a T10 basic ULTRA-TURRAX^®^ homogenizer (IKA-Werke GmbH and Co. KG, Staufen, Germany) on ice. The homogenates were then incubated on ice with shaking for 30 min and centrifuged at 10,000× *g* at 4 °C for 10 min. For the MPO assay, 0.1 mL of the resulting supernatant was added to 2.9 mL of reaction mixture. The reaction mixture consisted of 0.05 M phosphate buffer (pH 6.0), prepared by mixing equal volumes of 0.1 M phosphate buffer (50 mL) and *o*-dianisidine solution (16.64 mg in 50 mL of distilled water), supplemented with hydrogen peroxide solution (1.7 mL per 100 mL of total reaction mixture). Absorbance at 460 nm was measured for 5 min at 25 °C using a Shimadzu UV spectrophotometer. One unit of MPO activity was defined as the amount of enzyme required to convert 1 μmol of hydrogen peroxide per minute at 25 °C.

### 2.10. Hematoxylin and Eosin (H&E) Staining

For histological analysis, the collected tissue samples were fixed in 10% neutral-buffered formalin (FUJIFILM Wako Pure Chemical Corporation, Osaka, Japan), embedded in paraffin, and sectioned at a thickness of 4 μm. The tissue sections were then subjected to H&E staining to evaluate morphological changes. Briefly, the sections were deparaffinized in two changes in xylene for 5 min each and subsequently rehydrated through a descending ethanol series (100%, 95%, 90%, 80%, and 70%) for 2 min at each concentration, followed by rinsing in tap water for 10 min. The rehydrated sections were stained with Mayer’s hematoxylin solution (FUJIFILM Wako Pure Chemical Corporation) for 5 min at 20–25 °C, washed under running tap water for 10 min, and counterstained with eosin solution (Samchun Pure Chemical Co., Ltd., Pyeongtaek, Republic of Korea) for 1 min. After staining, the sections were dehydrated in 95% and 100% ethanol for 10 s each and cleared twice in xylene for 2 min each. Finally, the slides were mounted using Permount (Fisher Chemical, Waltham, MA, USA), coverslipped, and dried at 20–25 °C. The stained tissue sections were examined and imaged under a light microscope to assess histopathological changes.

### 2.11. Enzyme-Linked Immunosorbent Assay (ELISA) for Cytokine-Induced Neutrophil Chemoattractant-3 (CINC-3)

To quantify CINC-3 in the colonic lesions, harvested distal colon tissues were homogenized in potassium phosphate buffer (pH 6.0) using a T10 basic ULTRA-TURRAX^®^ homogenizer (IKA-Werke GmbH & Co. KG, Staufen, Germany) on ice. The homogenates were centrifuged at 10,000× *g* at 4 °C for 10 min, and the resulting supernatants were collected. CINC-3 levels were then measured using ELISA according to the manufacturer’s instructions (R&D Systems, Minneapolis, MN, USA).

### 2.12. Data Analysis

Statistical differences among experimental groups were assessed using one-way analysis of variance, followed by Tukey’s post hoc test for multiple comparisons. Ordinal colonic damage score (CDS) data were compared using the Mann–Whitney *U* test. A *p*-value of <0.05 was considered statistically significant. Quantitative data are presented as the mean ± standard deviation (SD).

## 3. Results

### 3.1. Synthesis of FBXT Conjugated with Acidic Amino Acids

**Figure 1:**
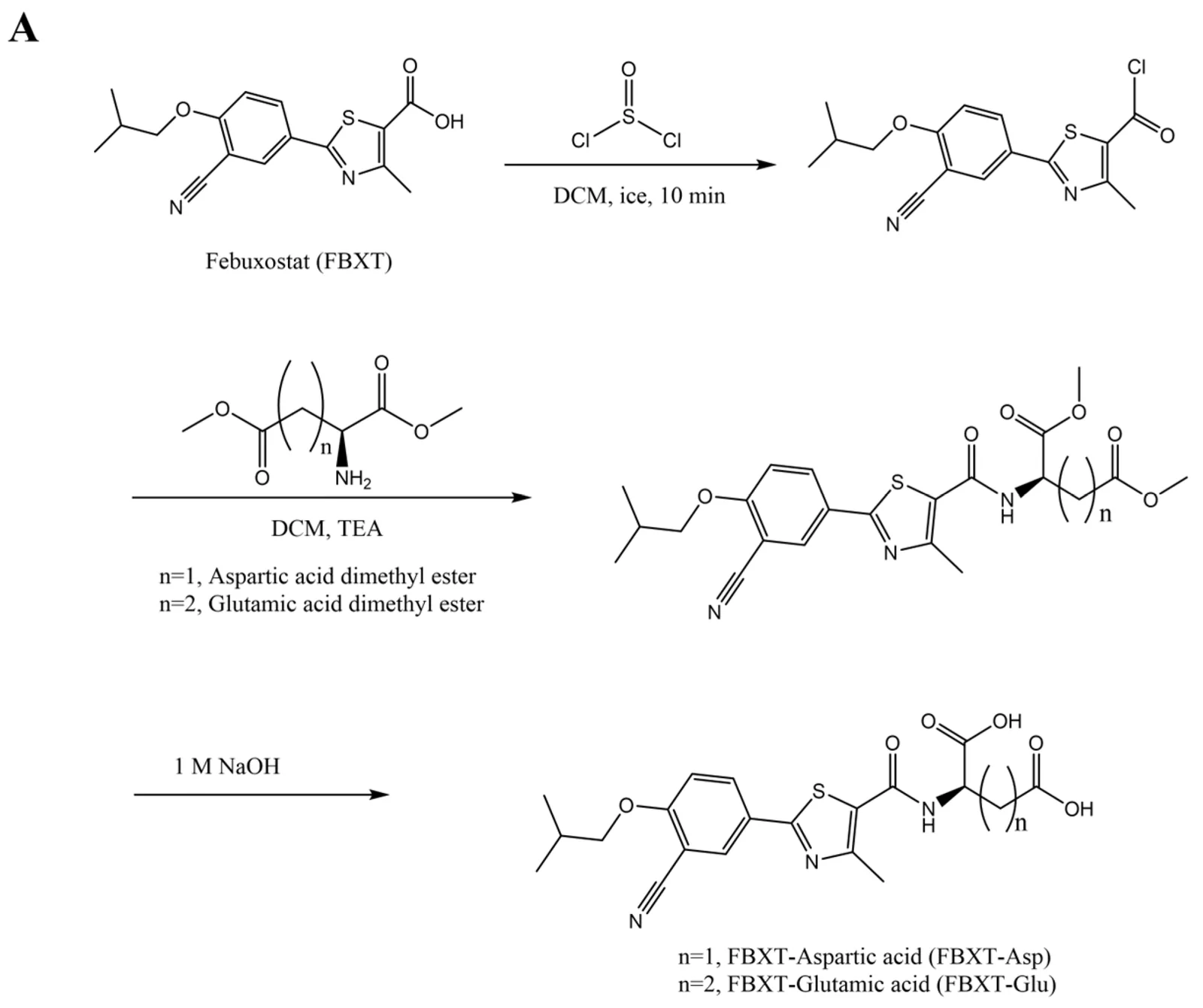

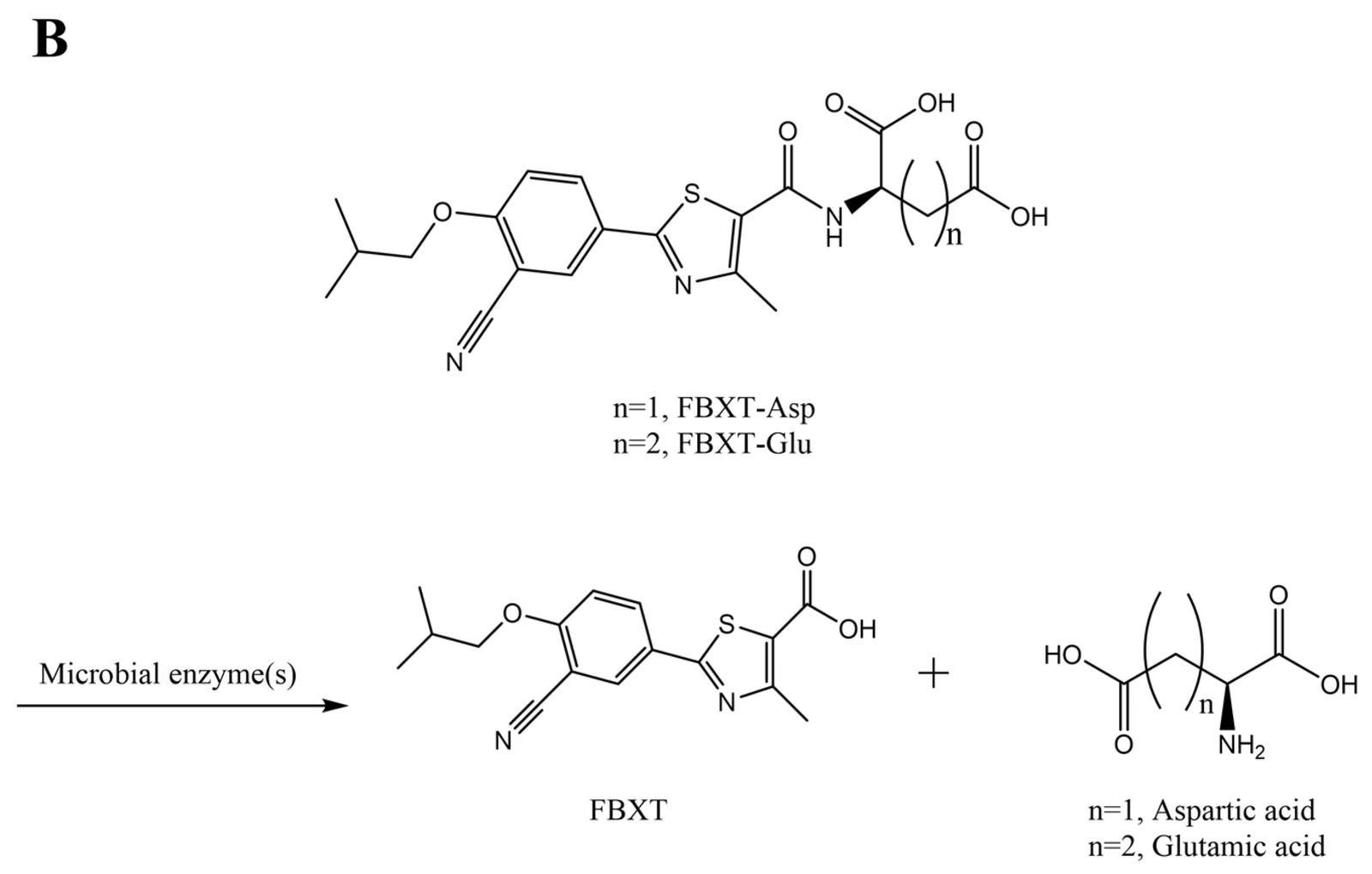
Synthesis, structural characterization, and colonic activation of febuxostat (FBXT) conjugates. (**A**) Synthesis of FBXT-Asp and FBXT-Glu. DCM—dichloromethane; TEA—triethylamine. (**B**) Proposed colonic activation of FBXT conjugates.

FBXT conjugates with acidic amino acids were synthesized as shown in Figure 1A. FBXT was conjugated with Asp and Glu through the formation of an amide bond between the carboxyl group of FBXT and the amino group of each acidic amino acid. The structures of the synthesized compounds were confirmed using FT-IR, ^1^H-NMR, and MS. In the FT-IR spectra of the conjugates, characteristic absorption bands attributable to the carbonyl groups of the amide (1645 cm^−1^ for FBXT-Asp and 1635 cm^−1^ for FBXT-Glu) and carboxylic acid (1698 cm^−1^ for FBXT-Asp [broad] and 1708 cm^−1^ for FBXT-Glu [broad]) groups were observed. The ^1^H-NMR spectra further supported conjugate formation, with characteristic signals corresponding to the amide protons detected at 8.5 ppm. Signals attributable to the aromatic and aliphatic protons of the conjugates were also observed. Moreover, molecular ion peaks corresponding to each conjugate were detected in the mass spectra. The FT-IR, ^1^H-NMR, and MS spectra are provided in Supplementary Information S5A–C, and the proposed colonic activation of the FBXT conjugates is shown in Figure 1B.

### 3.2. Colon Specificity of Acidic Amino Acid-Conjugated FBXT

**Figure 2:**
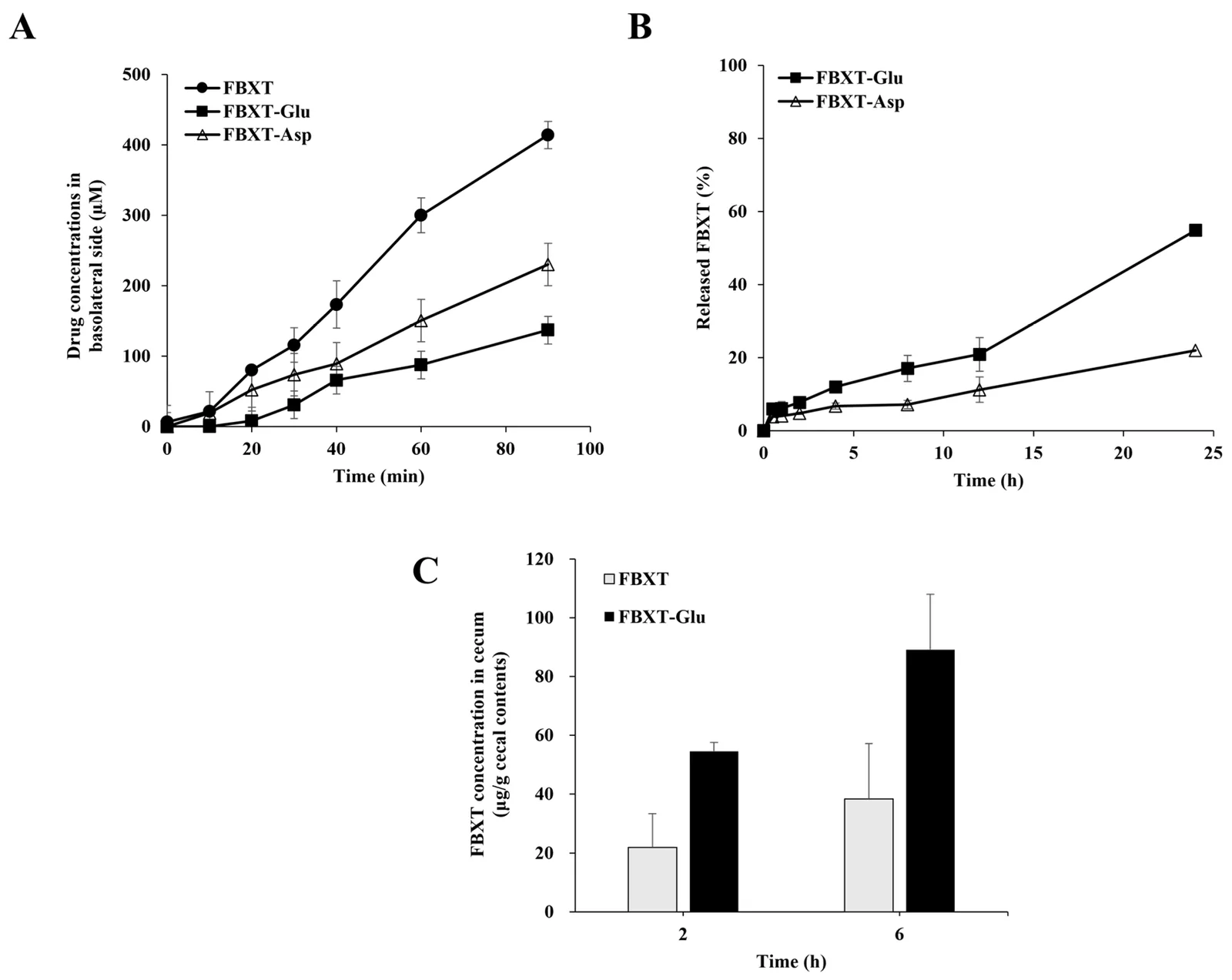
Colon-specific properties of FBXT conjugates. (**A**) Everted rat jejunal sacs were immersed in 8 mL of pre-warmed Krebs buffer containing 0.5 mM FBXT or its conjugates in 15-mL tubes and incubated for 90 min. The fluid levels inside and outside the sacs were carefully matched to prevent hydrostatic pressure differences. At predetermined time points, 50-μL aliquots were collected from the basolateral compartment, which initially contained 600 μL of Krebs buffer, and immediately replaced with an equal volume of fresh buffer. The amounts of transported drugs were quantified by HPLC. Data are presented as the mean ± SD (*n* = 3). (**B**) FBXT conjugates (0.5 mM) were incubated under nitrogen with a 10% (*w*/*v*) suspension of rat cecal contents in isotonic phosphate buffer (pH 6.8), and the release of FBXT was monitored over time by HPLC. Data are presented as the mean ± SD (*n* = 5). (**C**) After a 24-h fast with ad libitum access to water, male SpD rats (250–260 g) received FBXT (20 mg/kg) or FBXT-Glu (28 mg/kg, equivalent to 20 mg/kg FBXT) by oral gavage in 1.0 mL of PBS. The animals were euthanized at 2 and 6 h after administration, and cecal FBXT levels were quantified by HPLC. Data are presented as the mean ± SD (*n* = 5).

We evaluated the colon specificity of the acidic amino acid–FBXT conjugates by first assessing their stability during upper GI transit. The conjugates were incubated with rat small intestinal contents and in pH 1.2 buffer to assess their stability under intestinal and gastric conditions, respectively. Both conjugates remained intact in the small intestinal contents and acidic buffer for up to 10 h. The potential for systemic absorption of the conjugates was assessed by determining the distribution coefficients (log D_6.8_) and intestinal permeability of FBXT and its conjugates. Conjugation decreased the log D_6.8_ of FBXT from 1.451 to −1.217 for FBXT-Asp and −0.859 for FBXT-Glu, suggesting that the conjugates would undergo less efficient passive diffusion across the intestinal wall than FBXT. Consistent with this finding, intestinal permeability studies using everted rat intestinal sacs showed that FBXT transport across the intestinal wall was substantially reduced by conjugation with the amino acids (Figure 2A). FBXT transport was reduced by 43.7% and 64.3% following conjugation with Asp and Glu, respectively. To verify that these differences in transport were not attributable to drug-induced epithelial barrier damage, intestinal barrier integrity was assessed using FITC-dextran co-applied with either FBXT or its conjugates to the apical (donor) side under the same experimental conditions. FITC-dextran transport was minimal, with the basolateral concentration at 90 min corresponding to approximately 0.5% of the apical concentration, and did not differ among the control, conjugate-treated, and FBXT-treated groups. These findings indicated that the test compounds did not compromise intestinal barrier integrity. We next assessed whether the conjugates released FBXT in the large intestine by incubating them with rat cecal contents and monitoring FBXT release. To mimic the anaerobic environment of the large intestine, incubation with cecal contents was performed under nitrogen. As shown in Figure 2B, the conjugates underwent cleavage, resulting in the release of FBXT into the cecal contents. The conversion rates of FBXT-Asp and FBXT-Glu to FBXT were 7.1% and 17.0%, respectively, at 8 h and 22.0% and 54.8%, respectively, at 24 h. To investigate the involvement of microbial enzymes, additional incubations were performed using autoclaved cecal contents in which microbial enzymes had been inactivated [34]. No FBXT release was observed in the autoclaved cecal contents, supporting the involvement of microbial enzymes in conjugate cleavage. In vivo colon specificity was further evaluated by measuring cecal FBXT levels at 2 and 6 h after oral administration of either free FBXT or FBXT-Glu. FBXT-Glu was selected for the in vivo study because it exhibited lower intestinal transport and greater conversion to FBXT in the cecal contents than FBXT-Asp. As shown in Figure 2C, oral administration of FBXT-Glu resulted in greater cecal accumulation of FBXT than administration of free FBXT at both time points.

### 3.3. FBXT-Glu Potentiates the Anti-Colitis Activity of FBXT and Demonstrates Therapeutic Superiority over SSZ

**Figure 3:**
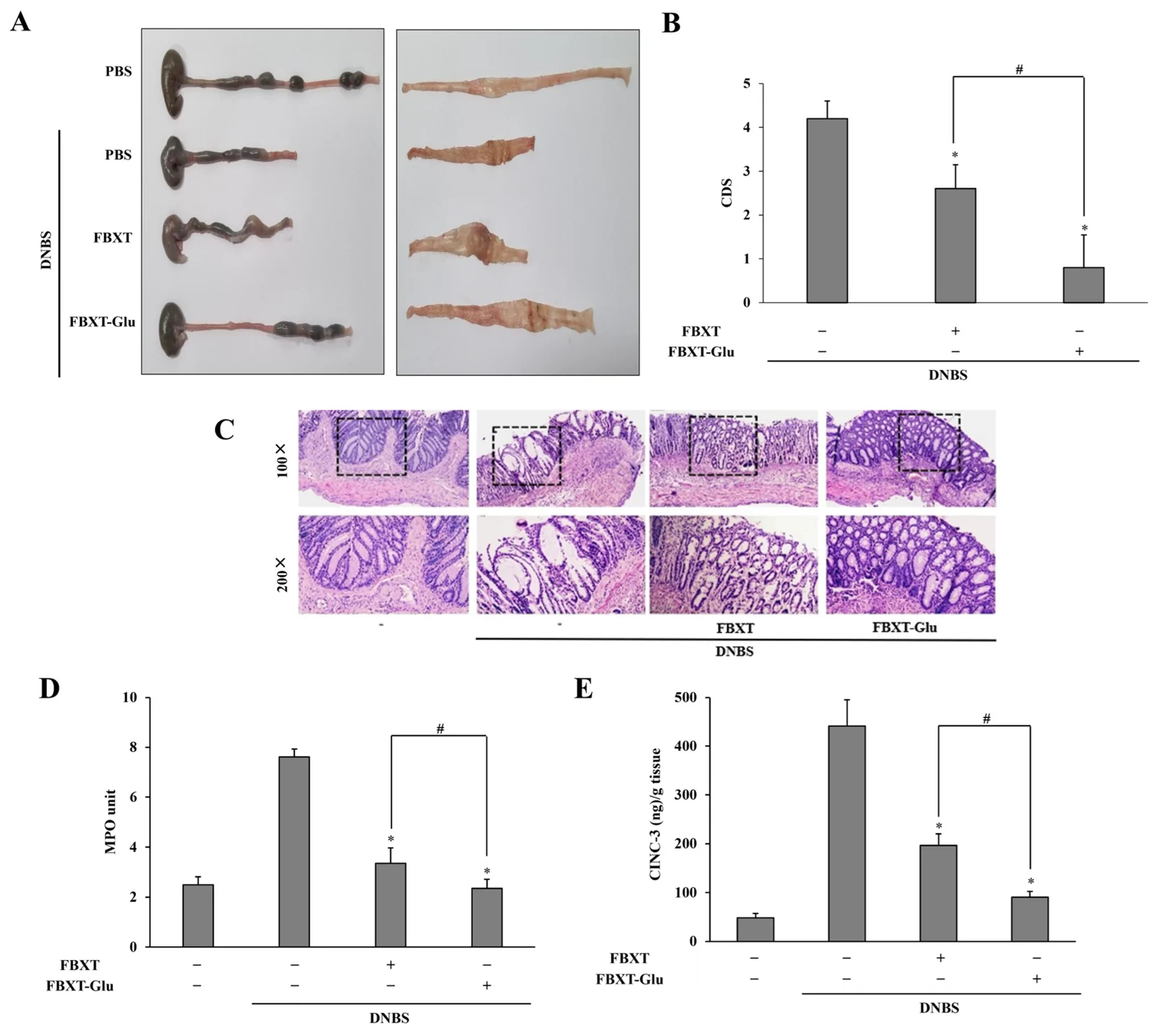

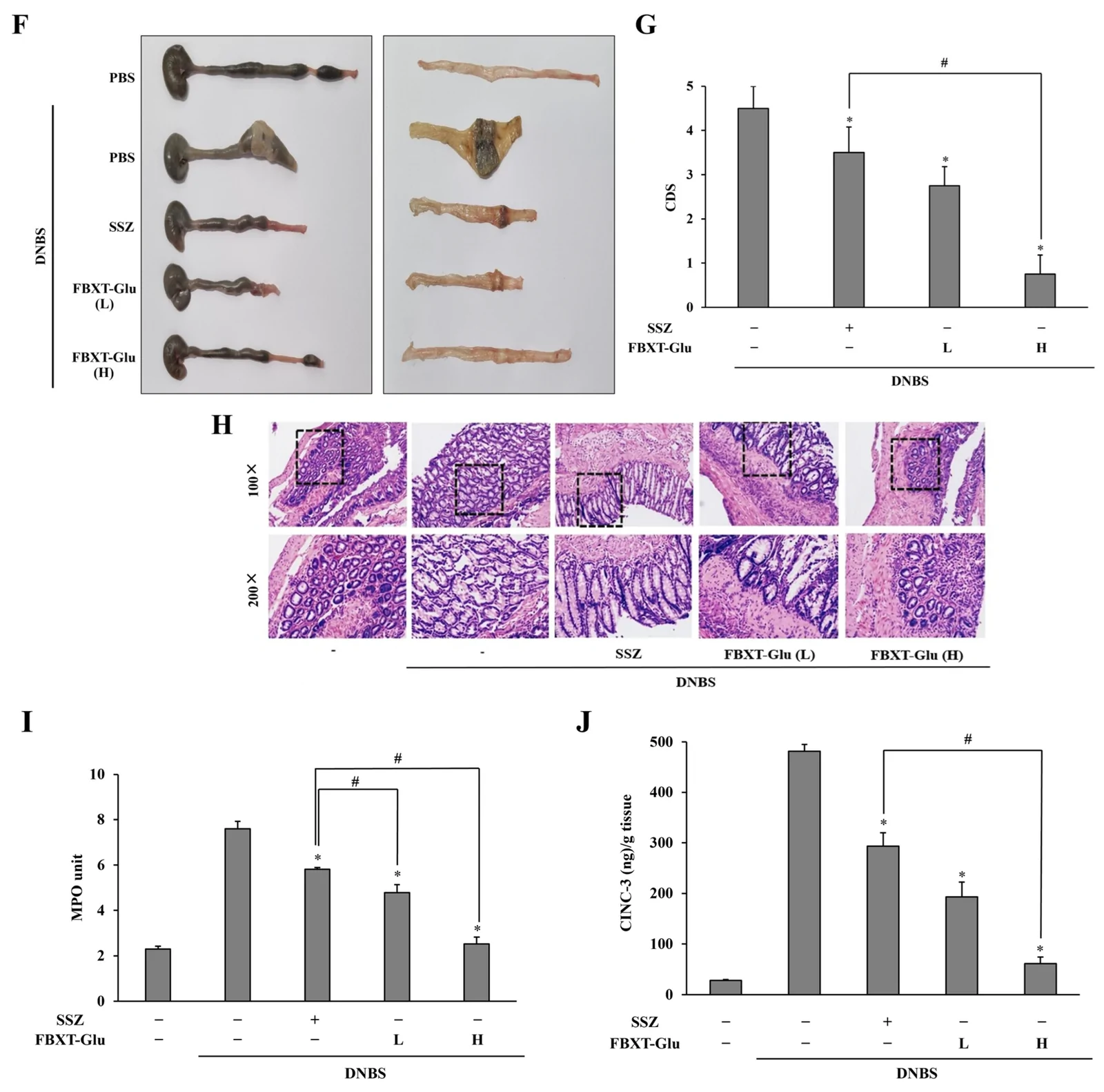
FBXT-Glu enhances the anti-colitis efficacy of FBXT and shows greater efficacy than SSZ in rats with DNBS-induced colitis. Rats with DNBS-induced colitis received FBXT (20 mg/kg) or FBXT-Glu (28 mg/kg, equivalent to 20 mg/kg FBXT) by oral gavage once daily for six consecutive days, starting 3 days after colitis induction. The drugs were administered as 1.0-mL suspensions in PBS, and tissue samples were collected 24 h after the final administration. (**A**) Representative images of the serosal and luminal surfaces of distal colon samples. (**B**) CDS for each treatment group. (**C**) Histological evaluation of colonic tissue by H&E staining. (**D**) MPO activity in the distal colon (4 cm). (**E**) Colonic CINC-3 levels determined through ELISA. Data are presented as the mean ± SD (*n* = 5). * *p* < 0.05 versus the DNBS-treated control; # *p* < 0.05. In an independent experiment, rats with DNBS-induced colitis received SSZ (100 mg/kg), low-dose (L) FBXT-Glu (14 mg/kg, equivalent to 10 mg/kg FBXT), or high-dose (H) FBXT-Glu (28 mg/kg, equivalent to 20 mg/kg FBXT) by oral gavage once daily for six consecutive days. The drugs were administered as 1.0-mL suspensions in PBS, and tissue samples were collected 24 h after the final administration. (**F**) Representative images of the serosal and luminal surfaces of distal colon samples. (**G**) CDS for each treatment group. (**H**) Histological evaluation of colonic tissue by H&E staining. (**I**) MPO activity in the distal colon (4 cm). (**J**) Colonic CINC-3 levels determined by ELISA. Data are presented as the mean ± SD (*n* = 5). * *p* < 0.05 versus the DNBS-treated control; # *p* < 0.05.

We investigated whether colonic delivery of FBXT enhanced its anti-colitis activity and compared its therapeutic efficacy with that of SSZ, a conventional colon-targeted 5-aminosalicylic acid prodrug. The rat doses of FBXT (10 and 20 mg/kg) corresponded to 1.25- and 2.5-fold, respectively, the human adult dose (80 mg/day), based on allometric dose scaling in which the rat dose was approximately sixfold the corresponding human dose [35]. In the first animal experiment, FBXT-Glu (at a dose equivalent to 20 mg/kg FBXT) or FBXT (20 mg/kg) was orally administered to rats with DNBS-induced colitis. Macroscopic examination of the colitis group revealed mucosal erosion hemorrhagic ulcers, luminal strictures, tissue edema, adhesion to adjacent organs, and colon shortening (Figure 3A). FBXT-Glu attenuated these macroscopic signs of colonic damage and resulted in a greater reduction in CDS than FBXT (Figure 3B). Consistent with these findings, H&E staining showed greater recovery of the colonic mucosa with FBXT-Glu than with FBXT (Figure 3C). FBXT-Glu also reduced colonic inflammation more effectively than FBXT, as indicated by reductions in MPO activity (Figure 3D) and levels of the inflammatory mediator CINC-3 (Figure 3E). In the second animal experiment, FBXT-Glu at doses equivalent to 10 (low dose [L]) and 20 (high dose [H]) mg/kg FBXT was orally administered to rats with DNBS-induced colitis, and its effects were compared with those of SSZ (100 mg/kg). FBXT-Glu dose-dependently attenuated macroscopic colonic damage (Figure 3F) and reduced CDS (Figure 3G), with the high dose producing greater effects than SSZ. H&E staining also showed greater recovery of the inflamed colonic mucosa with high-dose FBXT-Glu than with SSZ (Figure 3H). Furthermore, FBXT-Glu dose-dependently reduced colonic inflammation, as indicated by reductions in MPO activity (Figure 3I) and CINC-3 levels (Figure 3J). High-dose FBXT-Glu produced greater reductions in these inflammatory markers than SSZ.

### 3.4. Colonic Delivery of FBXT Reduces Its Systemic Absorption

**Figure 4:**
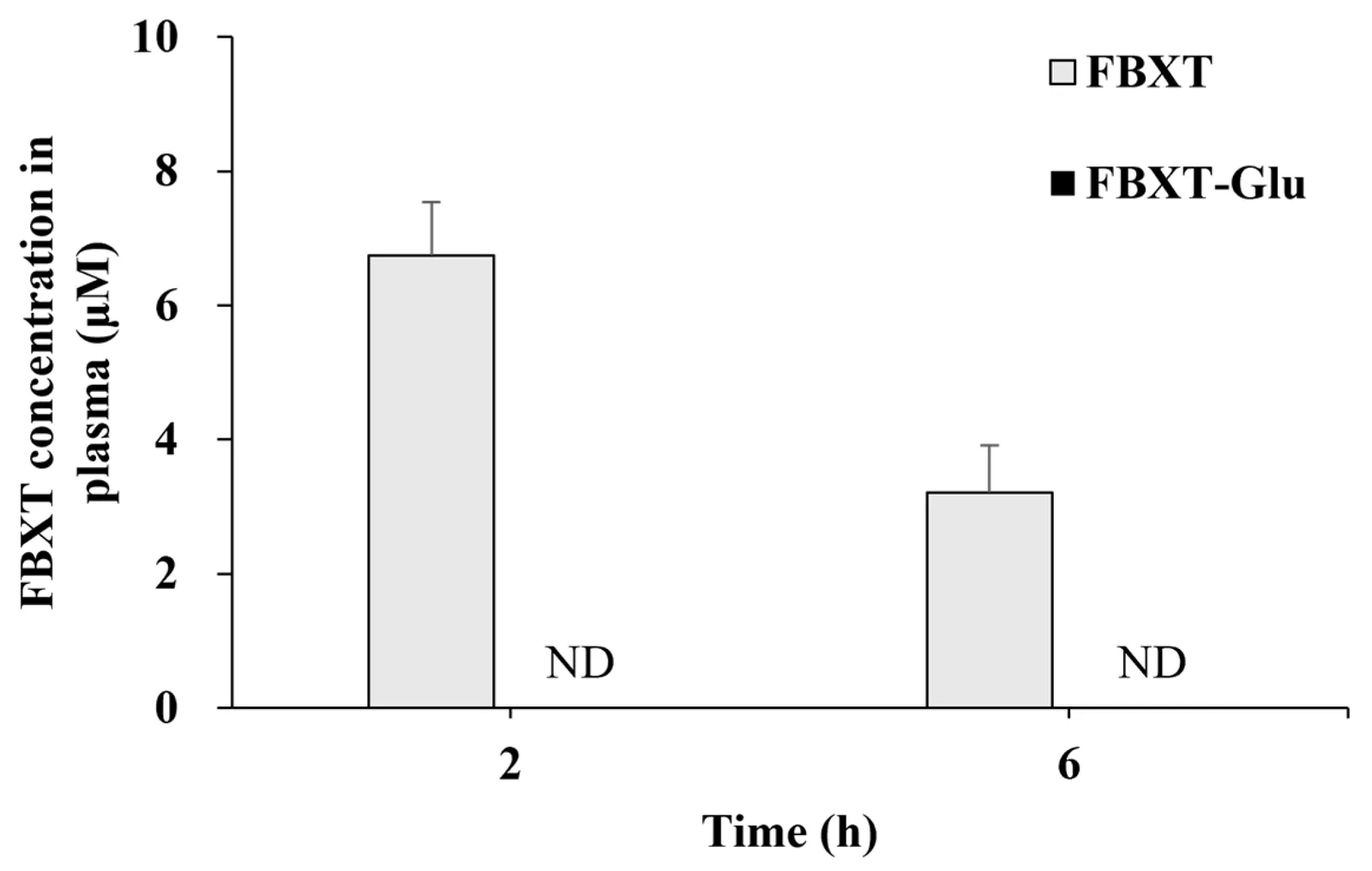
Colonic delivery of FBXT reduces its systemic absorption. After a 24-h fast with ad libitum access to water, male rats (250–260 g) were orally administered FBXT (20 mg/kg) or FBXT-Glu (28 mg/kg, equivalent to 20 mg/kg FBXT). At 2 and 6 h after administration, blood samples were collected via cardiac puncture, and plasma FBXT concentrations were determined using HPLC. Data are presented as the mean ± SD (*n* = 5). ND—not detectable (<1 μM).

Colon-targeted drug delivery generally reduces systemic absorption because the large intestine provides less favorable conditions for drug uptake, including a relatively small absorptive surface area, large luminal diameter, thick mucus layer, weak segmentation movements, and low water content [36,37]. To determine whether FBXT-Glu reduces the systemic absorption of FBXT, rats were orally administered FBXT or FBXT-Glu at equimolar doses, and plasma FBXT concentrations were measured at 2 and 6 h after administration. As shown in Figure 4, oral administration of FBXT resulted in a plasma concentration of approximately 6.7 μM at 2 h, whereas FBXT remained below the detection limit in plasma following oral administration of FBXT-Glu. These findings indicate that colonic delivery of FBXT not only enhances its anti-colitis efficacy but also minimizes systemic exposure, potentially reducing the risk of systemic adverse effects. Furthermore, plasma FBXT-Glu concentrations remained below the detection limit at the time points examined.

## 4. Discussion

In this study, FBXT was conjugated with acidic amino acids to achieve colon-specific delivery, and the anti-colitis efficacy of the selected conjugate was compared with that of SSZ. Our findings demonstrated that amino acid conjugation conferred colon-specific properties on FBXT. Among the conjugates, FBXT-Glu enhanced the anti-colitis efficacy of FBXT and showed greater efficacy than SSZ in rats with DNBS-induced colitis. Moreover, FBXT-Glu substantially reduced the systemic exposure to FBXT after oral administration, potentially reducing the risk of systemic adverse effects.

FBXT, which contains a carboxyl group, was conjugated with the acidic amino acids Asp and Glu via an amide linkage, an established approach for designing colon-specific prodrugs of carboxylic acid-containing drugs [30]. The introduction of two additional carboxyl groups (Figure 1A) increased the hydrophilicity of the conjugates. Consistent with this physicochemical change, amino acid conjugation reduced the passive transport of FBXT across the intestinal barrier, as demonstrated by the ex vivo everted rat intestinal sac assay (Figure 2A). In agreement with these findings, plasma FBXT reached approximately 6.7 μM at 2 h after oral administration of free FBXT, whereas FBXT remained below the detection limit following oral administration of FBXT-Glu. Together with the chemical and biochemical stability of the conjugates under conditions simulating the upper GI tract, as demonstrated by incubation in pH 1.2 buffer and with small intestinal contents, their limited intestinal absorption is expected to facilitate delivery of a substantial fraction of the administered conjugates to the large intestine. Upon reaching the large intestine, the conjugates can be converted to the parent drug FBXT through cleavage of the amide linkage, a process attributed to bacterial enzymes [30]. Consistent with this mechanism, the conjugates released FBXT during incubation with rat cecal contents, whereas no FBXT release was detected in autoclaved cecal contents. Furthermore, oral administration of FBXT-Glu resulted in greater cecal accumulation of FBXT than administration of free FBXT.

Consistent with previous studies [17,18,38], the XO inhibitor FBXT attenuated colonic damage and inflammation in rats with DNBS-induced colitis. Moreover, colon-targeted delivery appeared to enhance the anti-colitis activity of FBXT, as FBXT-Glu showed greater efficacy than free FBXT in this model. Therapeutic outcomes assessed via macroscopic examination, CDS determination, H&E staining, MPO activity measurement, and quantification of tissue levels of the potent proinflammatory chemokine CINC-3 consistently indicated greater anti-colitis effects of FBXT-Glu than of free FBXT. Furthermore, FBXT-Glu showed greater therapeutic efficacy than SSZ, with high-dose FBXT-Glu producing significantly greater reductions in colonic damage and inflammation in rats with DNBS-induced colitis.

FBXT is likely to coexist with intact FBXT-Glu at the target site for a certain period. Although FBXT-Glu may be too polar to readily reach intracellular FBXT targets, such as XO, by passive diffusion, it may potentially be taken up by colonic cells via peptide transporters [39]. Therefore, a direct contribution of intact FBXT-Glu to the overall anti-colitis effects cannot be completely excluded. However, evaluating the intrinsic anti-colitis activity of intact FBXT-Glu is challenging because of its conversion to FBXT at the target site. An uncleavable FBXT-Glu analog would be required to clarify the specific contribution of the intact conjugate to the observed therapeutic effects.

Beyond its enhanced therapeutic efficacy in the colon, FBXT-Glu may offer the additional advantage of minimizing potential systemic toxicity. Although free FBXT alleviated experimental colitis in rats, conventional oral administration resulted in substantial systemic absorption (Figure 4), potentially increasing hepatic and renal exposure [25,26]. Conversely, colon-specific delivery of FBXT via oral FBXT-Glu markedly reduced its systemic absorption, with plasma FBXT concentrations remaining below the detection limit at the time points examined (Figure 4). This reduced systemic exposure may enable localized treatment of colitis while limiting exposure of other tissues to FBXT. Although FBXT is clinically used as a long-term urate-lowering therapy for chronic gout [10,11,24], improving its safety profile while maintaining or enhancing its therapeutic efficacy for a new indication would provide an important advantage when repurposing FBXT as an anti-colitis agent.

The modest anti-inflammatory efficacy of mesalazine-based drugs, including SSZ, generally limits their clinical use to mild-to-moderate UC [40,41]. Given that FBXT-Glu showed greater anti-colitis efficacy than SSZ in the present rat model, colon-targeted FBXT may have potential as a therapeutic alternative to SSZ. With further preclinical and clinical validation, this approach may also warrant investigation in patients with more severe or mesalazine-refractory UC who require advanced therapies, such as biologics, corticosteroids, or immunomodulators [3,42].

Our findings support the therapeutic potential of colon-targeted FBXT as an anti-IBD strategy; however, further investigation is required before clinical translation. Its pharmacological efficacy should be validated in additional animal models, such as dextran sulfate sodium-induced colitis in rats or mice, which recapitulate pathological features of IBD distinct from those represented by the DNBS-induced rat model [43]. Additionally, the long-term local toxicity of colon-targeted FBXT at the site of action should be evaluated. Further elucidation of the mechanisms underlying its anti-colitis effects will also be important for supporting clinical translation.

In conclusion, acidic amino acid–FBXT conjugates show potential as colon-specific prodrugs of FBXT. FBXT-Glu may provide an orally administered anti-colitis therapy with greater efficacy than SSZ and a reduced risk of FBXT-related systemic adverse effects.

## Data Availability

All data generated or analyzed during this study are included in this published article and its Supplementary Information files.

## Supplementary Materials

The following supporting information can be downloaded at: https://www.mdpi.com/article/10.3390/pharmaceutics18091187/s1, Supplementary Information S1: The linear equations for the standard calibration of FBXT and its conjugates; Supplementary Information S2: Assessment of intestinal barrier integrity in the Jejunum; Supplementary Information S3: Scheme of animal experiments and treatment groups (A) Detailed scheme of animal experiments (B) Treatment groups in the first animal ex-periments (C) Treatment groups in the second animal experiments; Supplementary Information S4: Modified scoring system; Supplementary Information S5: (A) FT-IR spectra of FBXT, FBXT-Glu and FBXT-Asp. (B) ^1^H-NMR spectra of FBXT, FBXT-Glu and FBXT-Asp. (C) Mass spectra of FBXT-Glu and FBXT-Asp.

## Abbreviations

The following abbreviations are used in this manuscript:

^1^H-NMR	Proton nuclear magnetic resonance
Asp	Aspartic acid
AspDME	*L*-aspartic acid dimethyl ester
CD	Crohn’s disease
CDS	Colonic damage score
CINC-3	Cytokine-induced neutrophil chemoattractant-3
DCM	Dichloromethane
DMSO-d_6_	Deuterated dimethyl sulfoxide
DNBS	2,4-dinitrobenzenesulfonic acid
ELISA	Enzyme-linked immunosorbent assay
FBXT	Febuxostat
FITC	Fluorescein isothiocyanate
EA	Ethylacetate
ESI-MS	Electrospray ionization mass spectrometry
GI	Gastrointestinal
Glu	Glutamic acid
GluDME	*L*-glutamic acid dimethyl ester
H&E	Hematoxylin and eosin
HPLC	High-performance liquid chromatography
IBD	Inflammatory bowel disease
IL	Interleukin
MPO	Myeloperoxidase
PBS	Phosphate-buffered saline
ROS	Reactive oxygen species
SD	Standard deviation
SpD	Sprague–Dawley
SSZ	Sulfasalazine
TEA	Triethylamine
TNF-α	Tumor necrosis factor-alpha
UA	Uric acid
UC	Ulcerative colitis
XO	Xanthine oxidase
XOR	Xanthine oxidoreductase
