# Supplementary material for: Colon-Specific Delivery of Febuxostat via an Amino Acid Conjugate Enhances Anti-Colitis Efficacy and Limits Systemic Exposure
Source: Pharmaceutics. 2026 Sep 20;18(9):1187. doi: 10.3390/pharmaceutics18091187 (PMC13610556; doi:10.3390/pharmaceutics18091187)
Supplement: Supplementary file 1 [file pharmaceutics-18-01187-s001.zip › pharmaceutics-4538388-supplementary.pdf]

Supplementary Material

Colon-Specific Delivery of Febuxostat via an Amino Acid Conjugate Enhances Anti-Colitis Efficacy and Limits Systemic Exposure

Suji Kim[1], Sanghyun Ju[1], and Yunjin Jung[1, 2, *]

[1] College of Pharmacy, Pusan National University, Busan, 46241, Republic of Korea
[2] Research Institute for Drug Development, Pusan National University, Busan, 46241, Republic of Korea
* Correspondence: jungy@pusan.ac.kr; Tel.: +82-51-510-2527

Supplementary Information S1

The linear equations for the standard calibration of FBXT and its conjugates are listed below. In all the equations, y represents the peak area and x represents the drug concentration (μM).

1. Permeability assay

FBXT: y=16206x-710.96(R^2=0.9992)

FBXT-Glu: y=17505x-13589(R^2=0.9989)

FBXT-Asp: y=12605x-12857(R^2=0.9985)

2. Cecal contents (In vitro incubation)

FBXT: y=12025x-657.9(R^2=0.9982)

3. Pharmacokinetics (In vivo Cecum)

FBXT: y=1072.1x-208.78(R^2=0.9989)

4. Pharmacokinetics (In vivo Plasma)

FBXT: y=1599.1x+379.38(R^2=0.9998)

Supplementary Information S2

Assessment of intestinal barrier integrity in the Jejunum.

To verify that the drug transport was not due to epithelial barrier disruption intestinal barrier integrity was evaluated using FITC-dextran as a paracellular flux marker under the same experimental conditions as the transport study. Everted jejunal sacs were prepared as described in the main text. The basolateral side of each sac was filled with 600 μL of Krebs buffer, while the apical side contained 8 mL of the same buffer. FITC-dextran (100 μM) was added to the apical side together with either FBXT (0.5 mM) or its conjugates (0.5 mM). The sacs were incubated at 37 °C. Samples (50 μL) were collected from the basolateral side at 10, 20, 30, 40, 60, and 90 min and immediately replaced with an equal volume of fresh Krebs buffer to maintain a constant volume. The collected samples were mixed with 575 μL of methanol to precipitate proteins, followed by centrifugation. The supernatant was used for fluorescence analysis Fluorescence i ntensity was measured using a fluorescence spectrophotometer (excitation 495 nm, emission 520 nm). The apparent transport was expressed as the percentage of the FITC-dextran concentration on the basolateral side relative to the initial concentration on the apical side

A

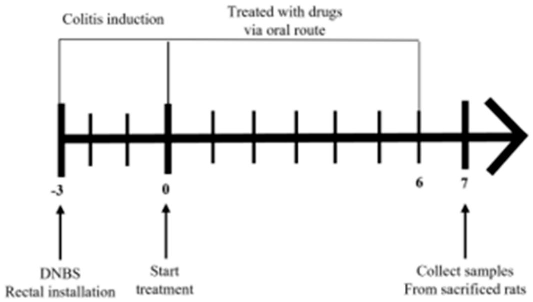

B

Group	Treatments via oral gavage
UT	Normal rats treated with vehicle only
DNBS	Colitis rats treated with vehicle only
FBXT	Colitis rats treated with FBXT (20 mg/kg)
FBXT-Glu	Colitis rats treated with FBXT-Glu (28 mg/kg)

C

Group	Treatments via oral gavage
UT	Normal rats treated with vehicle only
DNBS	Colitis rats treated with vehicle only
SSZ	Colitis rats treated with SSZ (100 mg/kg)
FBXT-Glu (L)	Colitis rats treated with FBXT-Glu (14 mg/kg)
FBXT-Glu (H)	Colitis rats treated with FBXT-Glu (28 mg/kg)

Supplementary Information S3. Scheme of animal experiments and treatment groups (A) Detailed scheme of animal experiments (B) Treatment groups in the first animal experiments (C) Treatment groups in the second animal experiments

Supplementary Information S4. Modified scoring system

Score	Features
0	No detectable macroscopic changes (Normal appearance)
1	Localized tissue hyperemia without any ulcer formation
2	Linear ulcerations displaying negligible acute inflammation
3	Inflammatory lesions and ulcerated areas spanning 2–4 cm
4	Serosal adhesion to adjacent organs, accompanied by 2–4 cm of inflammation and ulceration
5	Severe stricture, extensive serosal adhesions across multiple bowel loops, or inflammation and ulceration exceeding 4 cm

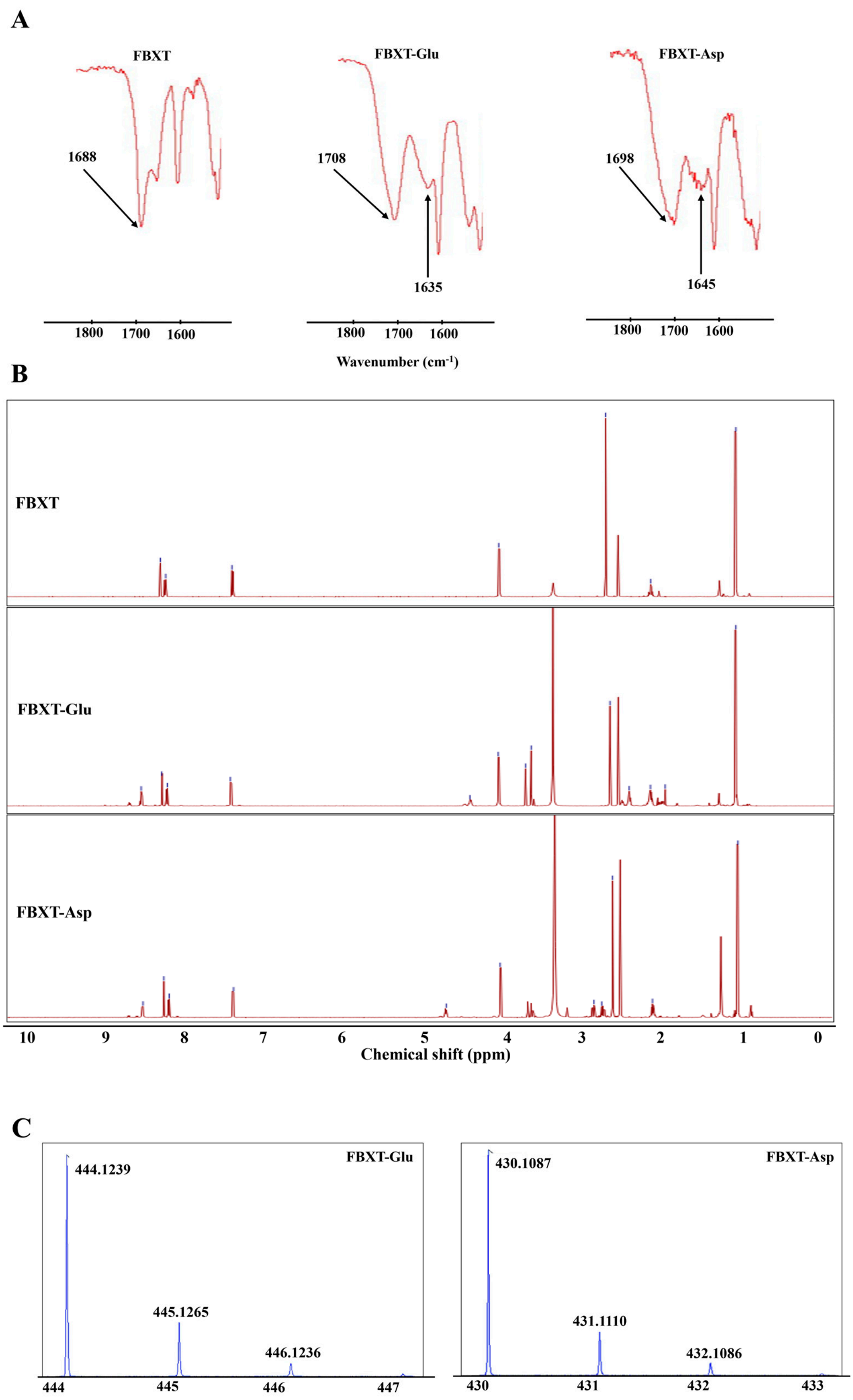

Supplementary Information S5. (A) FT-IR spectra of FBXT, FBXT-Glu and FBXT-Asp. (B) ^{1}H-NMR spectra of FBXT, FBXT-Glu and FBXT-Asp. (C) Mass spectra of FBXT-Glu and FBXT-Asp.
